# Supplementary figures and images for: Age Deceleration and Reversal Gene Patterns in Dauer Diapause
Source: Aging Cell. 2025 Oct 17;24(12):e70253. doi: 10.1111/acel.70253 (PMC12686545; doi:10.1111/acel.70253)

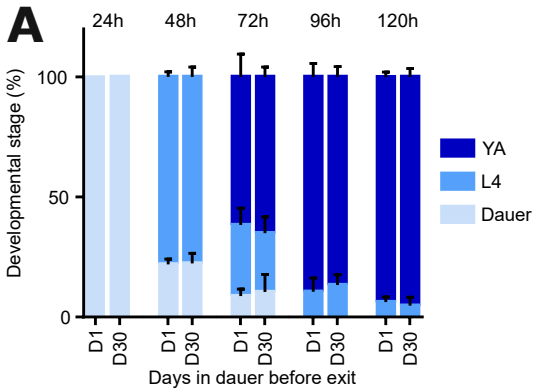

Supplement: Supplementary file 1 — Figure S1: Developmental resumption assay of daf‐2 animals arrested in dauer for 1 or 30 days. At each indicated timepoint after diapause exit induction, the percentage of animals in dauer (gray), L4 (light blue), or young adult (YA, dark blue) stage is shown. One representative experiment is shown. [file ACEL-24-e70253-s006.pdf]

**A**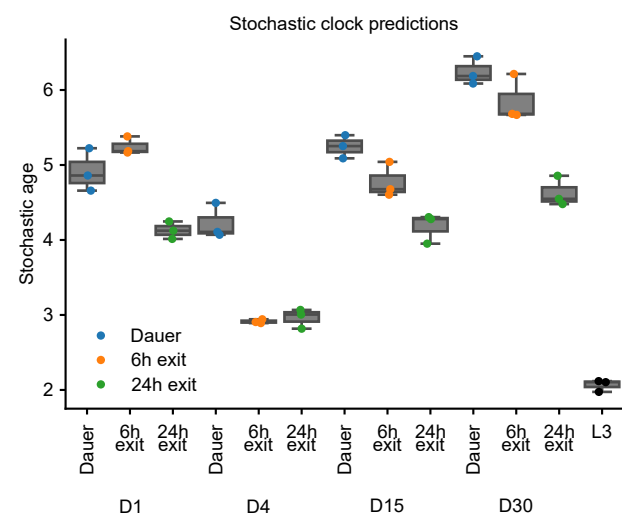**B**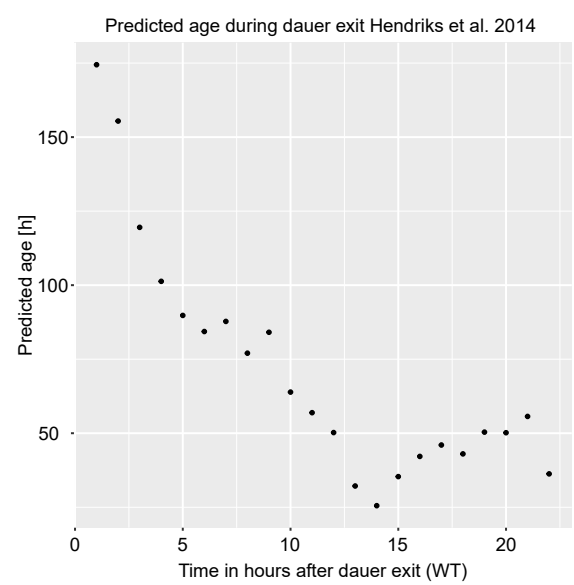**C**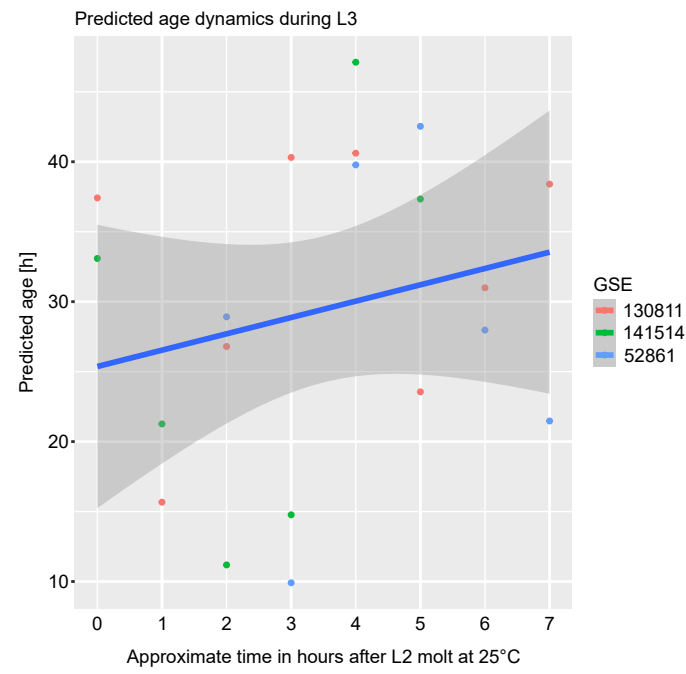

Supplement: Supplementary file 2 — Figure S2: (A) Predicted age for dauer and L3 samples using the stochastic age transcriptomic clock. Each dot represents a single RNA‐seq sample. The x‐axis nomenclature features first the respective day, followed by the number of hours post‐exit after the dot. (B) BiT Age predictions for biological age of the dauer exit time series dataset of Hendriks et al. (2014). (C) Predicted biological age of samples in the L3 stage using the BiT Age transcriptomics clock. The GSE ID of the public dataset is color‐coded. [file ACEL-24-e70253-s007.pdf]

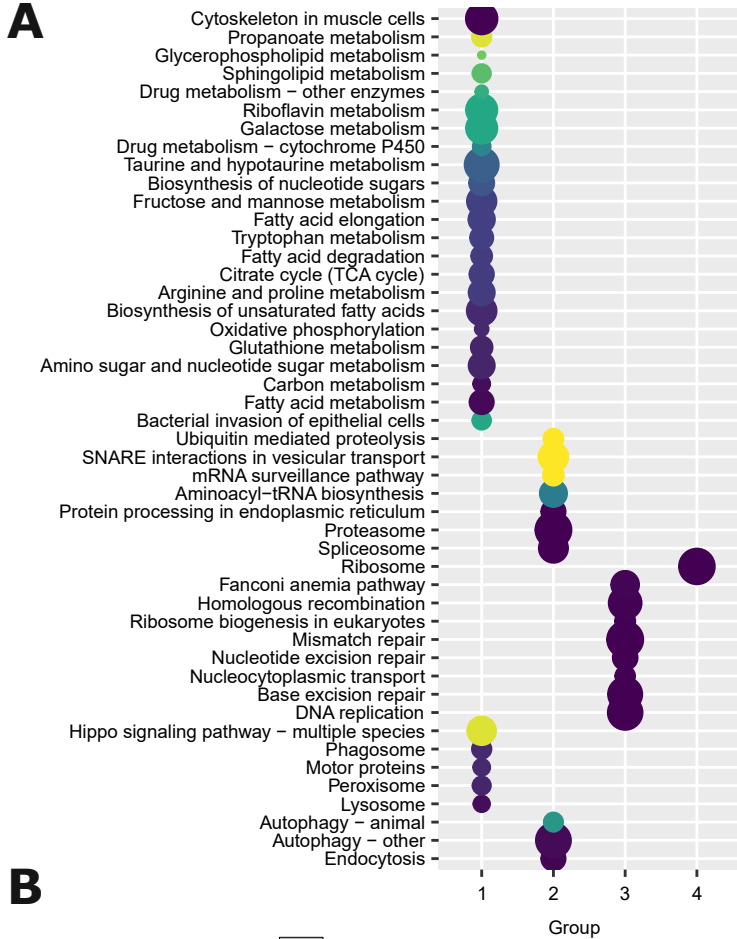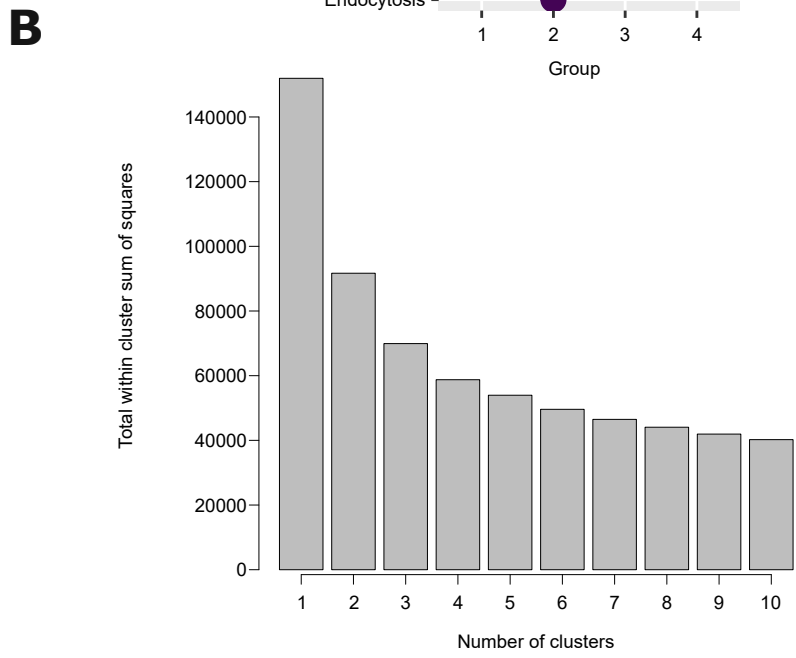

Supplement: Supplementary file 3 — Figure S3: (A) Shown are all significantly enriched KEGG pathways for each of the four gene clusters identified across the dauer aging time course. The x‐axis represents the gene clusters (Groups 1–4), while the y‐axis lists all enriched pathways. Color‐coded is the adjusted p value, the bubble size shows the ratio of differentially expressed genes in a pathway to the total number of genes in the pathway. This figure expands on the subset of pathways presented in Figure 3C. (B) The plot shows the total within‐cluster sum of squares (WCSS) for k‐means clustering across different values of k (number of clusters) on the x‐axis. As the number of clusters increases, the WCSS decreases, indicating improved clustering fit. The “elbow” point suggests the optimal number of clusters. In this case, the elbow occurs at k = 4, indicating that a four‐cluster solution provides a good balance between explanatory power and model simplicity. [file ACEL-24-e70253-s004.pdf]

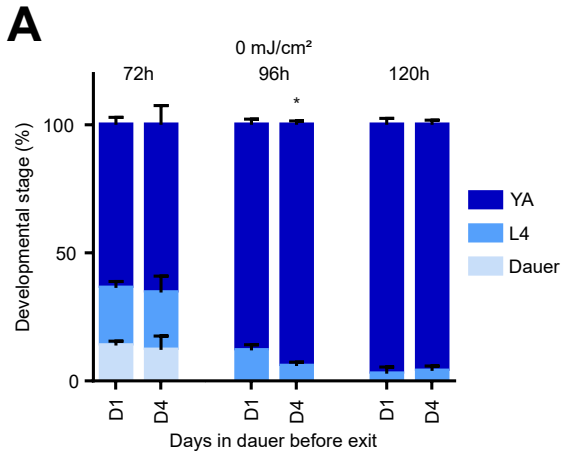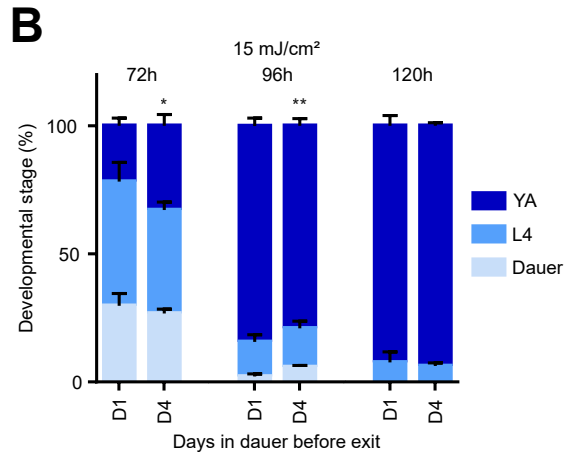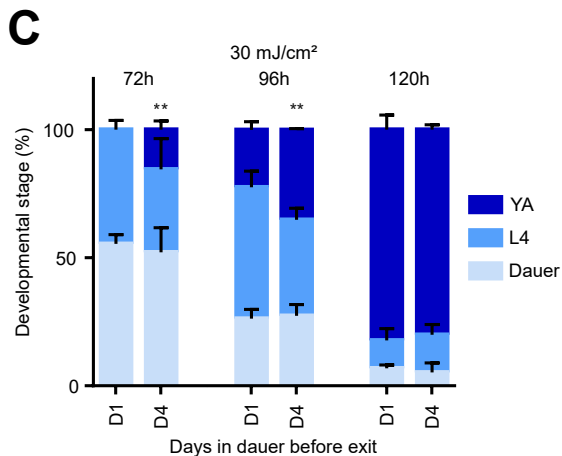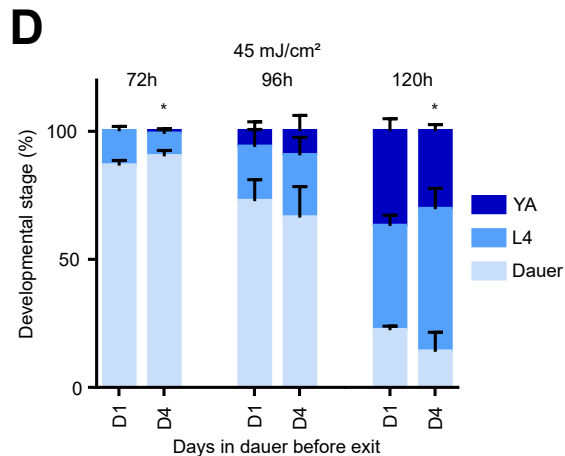

Supplement: Supplementary file 4 — Figure S4: Dauer exit is similar between D1 and D4 UV‐treated dauers. daf‐2 animals were UV‐ or mock‐treated at day 1 of dauer. Panels (A) to (D) show, respectively, 0, 15, 30 and 45 mJ/cm2 conditions. Dauer exit was induced immediately after UV treatment. Average of n = 3 independent experiments per dose is shown, error bars represent the standard deviation (SD). Two‐tailed t‐test. (*p ≤ 0.05, **p ≤ 0.01). [file ACEL-24-e70253-s003.pdf]

**A**

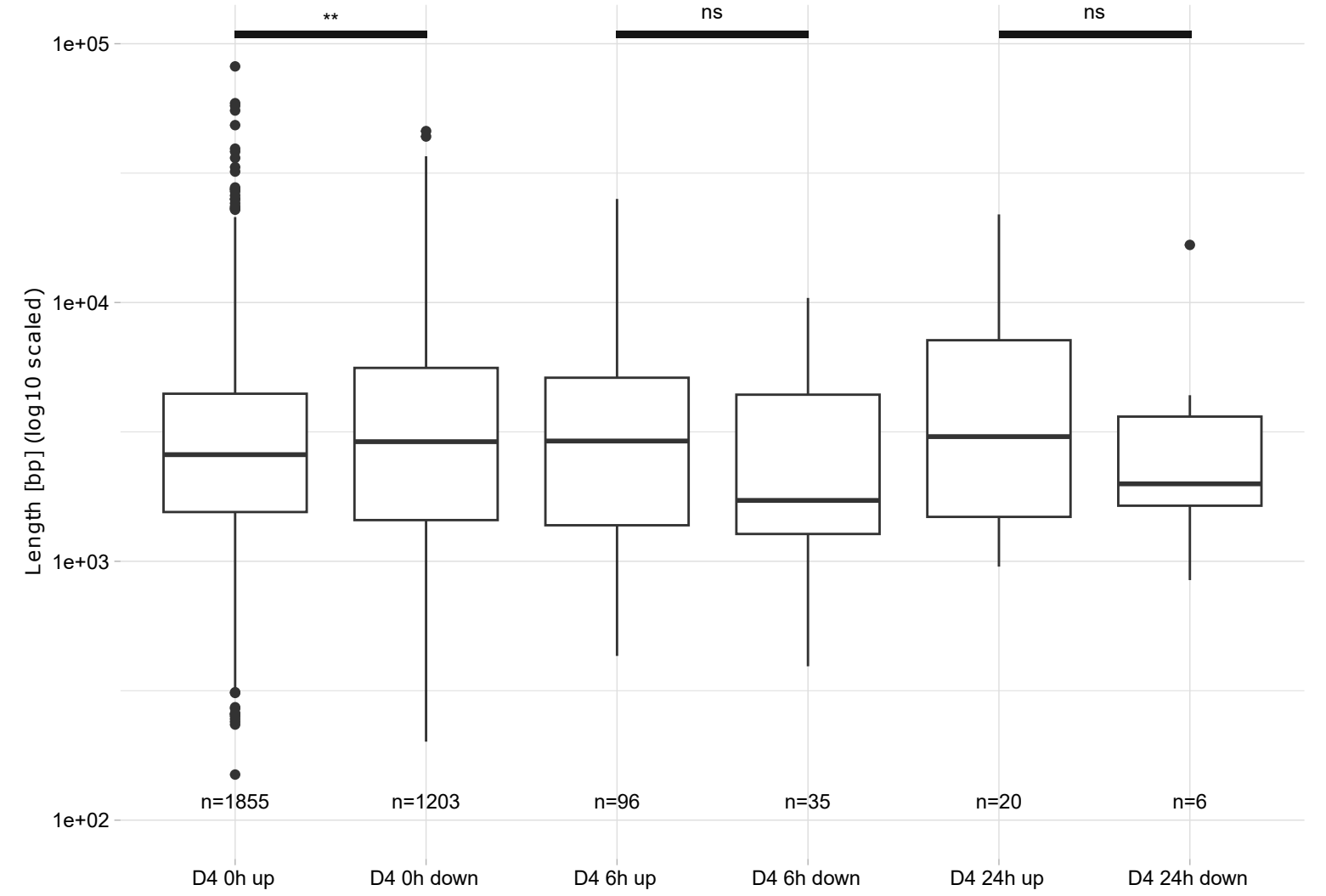

**B**

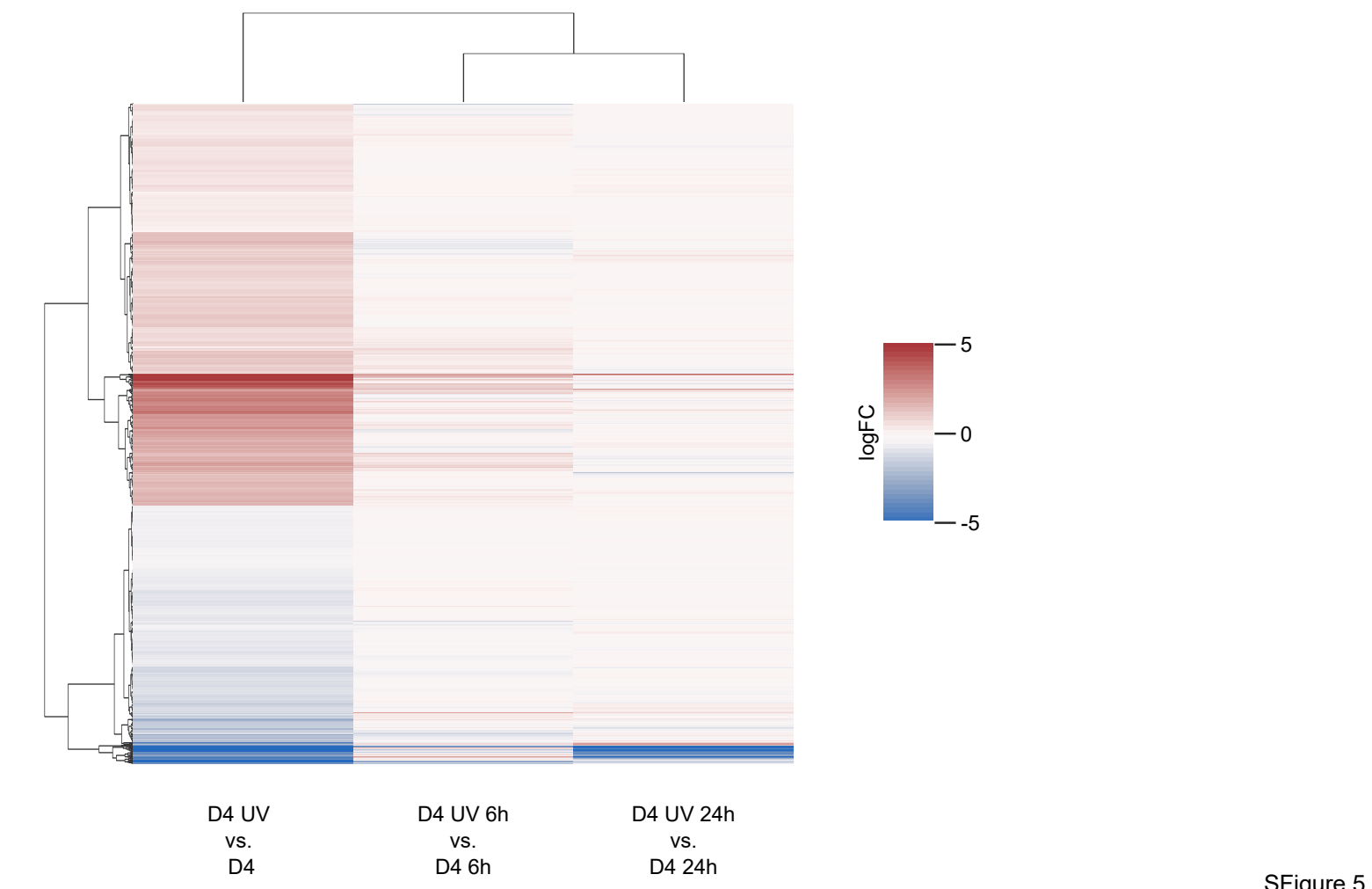

Supplement: Supplementary file 5 — Figure S5: (A) Box plots of gene lengths of genes differentially up‐ or down‐regulated in UV‐treated samples of dauer exit on D4 relative to untreated samples of dauer exit on D4 with n being the number of genes in the set. Mann–Whitney U test was used to identify statistically significant differences in gene lengths between the groups. (*p < 0.05, **p < 0.01, ***p < 0.001). (B) Heatmap showing the log2 fold change (logFC) of genes that are significantly differentially expressed (FDR < 0.05) in at least one of the three comparisons: D4 UV versus D4, D4 UV 6 h versus D4 6h, and D4 UV 24 h versus D4 24h. Rows represent individual genes, and columns represent the pairwise comparisons. Genes were clustered using hierarchical clustering with the Ward method and Euclidean distance. The color scale indicates the direction and magnitude of differential expression (blue: downregulated; red: upregulated). [file ACEL-24-e70253-s005.pdf]
